# Autonomic dysfunction in posttraumatic stress disorder indexed by heart rate variability: a meta-analysis

**DOI:** 10.1017/S003329172000207X

**Published:** 2020-09

**Authors:** Martha Schneider, Andreas Schwerdtfeger

**Affiliations:** Institute of Psychology, University of Graz, Graz, Austria

**Keywords:** Heart rate, heart rate variability, meta-analysis, posttraumatic stress disorder

## Abstract

**Background:**

Changes in autonomic nervous system (ANS) function have been observed in a variety of psychological disorders, including posttraumatic stress disorder (PTSD). Analysis of heart rate variability (HRV) provides insight into the functioning of the ANS. Previous research on PTSD found lower HRV in PTSD patients compared to controls, indicating altered sympathetic and parasympathetic activity, but findings are inconsistent. The purpose of this meta-analysis was to examine differences in HRV indices between individuals with PTSD and healthy controls at baseline and during stress.

**Methods:**

The included primary studies present an aggregate of studies analyzing different HRV indices. Examined HRV indices were standard deviation of the normalized NN-intervals (SDNN), root mean square of successive differences (RMSSD), low-frequency (LF) and high-frequency (HF) spectral components, LF/HF ratio, and heart rate (HR). Moderating effects of study design, HRV and PTSD assessment, and sample characteristics were examined via subgroup-analyses and meta-regressions.

**Results:**

Random-effects meta-analyses for HRV parameters at rest revealed significant group differences for RMSSD and HF-HRV, suggesting lower parasympathetic activity in PTSD. The aggregated effect size for SDNN was medium, suggesting diminished total variability in PTSD. A small effect was found for LF-HRV. A higher LF/HF ratio was found in the PTSD sample as compared to controls. Individuals with PTSD showed significantly higher HR. During stress, individuals with PTSD showed higher HR and lower HF-HRV, both indicated by small effect sizes.

**Conclusions:**

Findings suggest that PTSD is associated with ANS dysfunction.

## Introduction

Exposure to traumatic life events can have severe effects on the functioning of physiological systems, including the autonomic nervous system (ANS) (Orr & Roth, 2000). The cardiovascular system, digestive tract, respiratory system and the regulation of the metabolism are controlled and regulated by the ANS (Jänig, 2008). The ANS consists of two branches, the sympathetic nervous system (SNS), and the parasympathetic nervous system (PNS). Both branches are interconnected and work mostly unconsciously (Johnson, 2018). The SNS can boost body performance during increased activity or stress, which is indicated by heightened blood pressure, heart rate and respiration (Murison, 2016). The PNS is responsible for storing and building up energy during periods of rest or recovery. During PNS activity, heart rate slows down and blood pressure lowers (Pichon & Chapelot, 2010). A balanced interaction of both systems is thought to enable an individual's adaptive adjustment to changing environments (Elliott & Lawrenson, 2009).

Dysregulations in the ANS are thought to characterize a variety of psychological disorders, including posttraumatic stress disorder (PTSD) (Minassian et al., 2015). Symptoms of intrusion, avoidance, negative cognitions and mood, and alterations in arousal and reactivity, characterize PTSD (American Psychiatric Association, 2013). Alterations in arousal and reactivity suggest ANS abnormalities in PTSD patients. Yehuda, Southwick, Giller, Ma, and Mason (1992) reported higher levels of dopamine and norepinephrine concentrations in PTSD patients, which might contribute to changes in ANS activity. Furthermore, a meta-analysis by Pole (2007) revealed that resting heart rate is increased in patients with PTSD as compared to healthy controls. Alterations in arousal may also change cardiovascular stress activity and reactivity. This can lead to either increased or blunted responsiveness of individuals with PTSD to challenging and stressful tasks (Cohen et al., 2000; Dennis et al., 2016; Jovanovic, Norrholm, Sakoman, Esterajher, & Kozarić-Kovačić, 2009).

Analysis of heart rate variability (HRV) allows to gain more insight into the functioning of the ANS (for an overview, see Schwerdtfeger et al., 2020). HRV is based on the beat to beat variations in the heart rate, which are caused by varying influences of the SNS and the PNS. HRV parameters are derived from electrocardiogram signal analysis, and can be quantified using time-domain, frequency-domain and non-linear methods. Root mean square of successive differences (RMSSD) and standard deviation of NN intervals (SDNN) are common time-domain parameters. High-frequency HRV (HF-HRV; 0.15–0.40 Hz), low-frequency HRV (LF-HRV; 0.04–0.15 Hz) and the LF/HF ratio are commonly examined frequency domain-parameters. Mechanisms of the cardiovascular regulation may also interact in a non-linear manner. Approximate entropy and detrended fluctuation analysis quantify short-term HRV (Camm et al., 1996). Non-linear analysis of HRV, however, has been less often conducted, and its benefits relative to the traditional time and frequency domain measures are under debate (de Godoy, 2016; Voss, Schulz, Schroeder, Baumert, & Caminal, 2008).

RMSSD and HF-HRV are highly intercorrelated and associated with parasympathetic (i.e. vagal) activity (Thayer & Lane, 2007). Both, SNS and PNS activities influence SDNN, which displays overall flexibility of the ANS (Shaffer & Ginsberg, 2017). Influence of the SNS and PNS on LF-HRV is discussed controversially. Recent research showed that LF-HRV might be associated with baroreceptor activity, suggesting that LF-HRV might primarily reflect parasympathetic activity (Goldstein, Bentho, Park, & Sharabi, 2011). High HRV is a sign of good adaptability of the cardiovascular system, which enables an individual to adapt to inner and outer changes (McCraty & Shaffer, 2015; Schwerdtfeger et al., 2020). Overactivation of the SNS and decreased PNS activity may cause low HRV. Low HRV is a maker of impaired health (Thayer & Lane, 2007), and a risk factor for the onset of cardiovascular disease (Hillebrand et al., 2013). Therefore, lower HRV might impose risk for the development of secondary cardiovascular diseases in patients with PTSD.

Importantly, previous meta-analyses on HRV and PTSD suggest that HRV is lower in PTSD patients compared to controls (Campbell, Wisco, Silvia, & Gay, 2019; Nagpal, Gleichauf, & Ginsberg, 2013). Campbell et al. (2019) focused on differences in HRV parameters, which mainly reflect parasympathetic activity. Several vagally-mediated HRV parameters were combined, leading to one average effect size per study. Results revealed lower vagally-mediated HRV in individuals with PTSD compared to controls (Hedges' *g* = −0.26). Nagpal et al. (2013) analyzed HRV parameters separately. The aggregated effect sizes for HF-HRV, LF-HRV, LF/HF, RMSSD and SDNN were quite large (range Hedges' *g*: −0.33 to −2.94), probably resulting from a lower number of studies included in the meta-analyses and the exclusion of unpublished studies. HRV parameters were lower in PTSD patients compared to controls, indicated by medium to large effect sizes. The largest effect was found for HF-HRV (Hedges' *g* = −2.27). Both studies reported moderate to high levels of heterogeneity though.

The aim of the current meta-analysis was to examine differences in HRV parameters between individuals with PTSD and controls, both at rest and during stress tasks. In contrast to the meta-analysis of Campbell et al. (2019), which combined interrelated measures of HRV, the current meta-analysis will provide separate results for specific HRV parameters. This approach was chosen, since there is no consensus regarding treating interrelated HRV measures equivalent (Shaffer & Ginsberg, 2017). Separate analysis of HRV parameters should lead to a differentiated understanding of alterations in HRV in PTSD. Furthermore, a more complete picture of the relation between PTSD and HRV will be provided by including unpublished studies and LF-HRV. Additionally, moderator variables will be examined that may influence differences in HRV alterations in PTSD and controls. Finally, a new method to verify and control for publication bias will be applied, which allows for correction of effect size estimates of publication bias in the presence of heterogeneity in true effect sizes. Analyzing effect sizes for different HRV parameters both at rest and during stress will contribute to a deeper understanding of the adaptive capacity in individuals suffering from PTSD.

## Methods

The current meta-analysis was conducted in accordance to the ‘Preferred Reporting Items for Systematic Reviews and Meta-Analysis (PRISMA)’ guidelines (Moher et al., 2015).

### Literature search and inclusion criteria

**Fig. 1.**
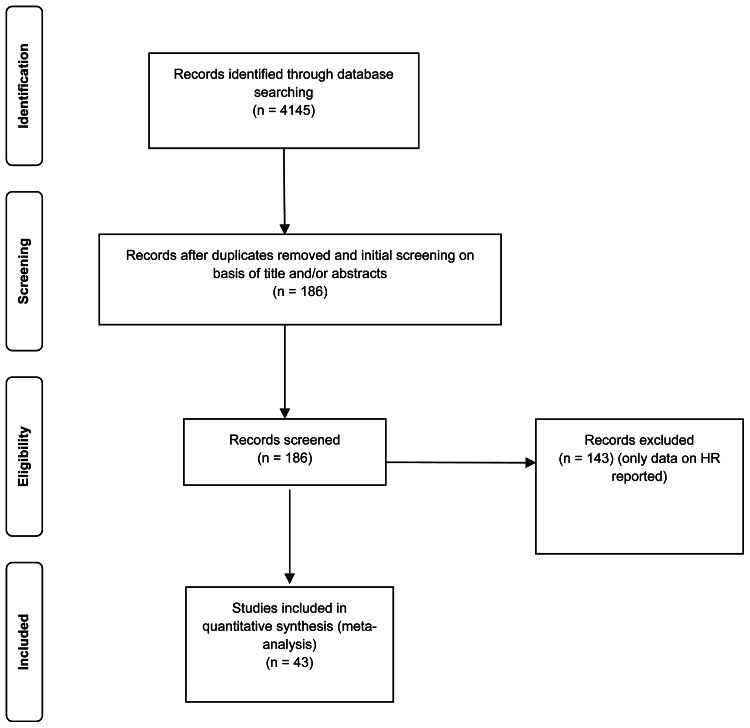
PRISMA flow diagram.

A systematic literature search was performed up to March 2019. Databases used to identify relevant articles were: PubMed, PsycInfo, Cinahl, Web of Science and Google Scholar. The following search terms were used: ((post traumatic stress disorder) OR (post traumatic stress) OR (posttraumatic stress disorder) OR (PTSD)) AND ((heart rate variability) OR (HRV) OR (respiratory sinus arrhythmia) OR (RS) OR (heart period variability) OR (RR variability) OR (cycle length variability) OR (vagal nerve activity) OR (sinus arrhythmias) OR (autonomic nervous system) OR (parasympathetic nervous system) OR (sympathetic nervous system) OR (vagus nerve)). Reference lists of articles included in the meta-analysis were browsed for additional studies. Unpublished data were retrieved by contacting researchers, who had already published data on PTSD and HRV. Additionally, effect sizes of unpublished studies were collected through supplemental material of Campbell et al.'s (2019) meta-analysis. Time period restrictions or language restrictions were not set. Details on study search and selection process are shown in Fig. 1. Studies were included in the meta-analysis if they (1) reported a measure of PTSD, (2) reported a measure of HRV and (3) included only adult samples (⩾18 years). Studies were excluded if they did not report quantitative data necessary for effect size calculation. Case studies and systematic reviews were excluded as well.

### Data extraction

A data extraction sheet was generated to collect data based on inclusion criteria and common study characteristics. Throughout the process of study extraction, the sheet was continuously adapted. The data extraction sheet included basic study information (author, publication year and published *v*. unpublished data), details on PTSD (sample size, DSM measure and PTSD measure), details on HRV (measure of HRV, laboratory or ambulatory assessment, recording position, length of measurement and sampling rate) and information on moderators. HRV parameters for baseline and – if available – for stress conditions were extracted. Data on heart rate (HR) were extracted as a secondary measure of HRV. Data on HR were only extracted in case the primary study included measures on HRV. In case of a stressor task, baseline was defined as the HRV recording time before the stressor occurred. Reported standard errors were transformed to standard deviations for effect size estimation, using the following formula: s.d. = s.e. × √*n* (Higgins & Green, 2011). In case of missing standard errors for reported effect size from categorical studies, the following approach was used: to convert from Cohens' *d* to Hedges' *g* a correction factor was used, called *J*. An approximation was used for *J* (*J* = 1 − (3/4*v* − 1). Hedges' *g* was transformed to Cohens' *d* (*g* = *J* × *d*). The variance for Cohens' *d* was calculated (*V*_d_ = *n*_1_ + *n*_2_/*n*_1_ × *n*_2_ + *d*^2^/2(*n*_1_ + *n*_2_)). Sampling variance of *g* was derived from back converting (*V*_g_ = *J*^2^*V*_d_). Standard error was derived via square root. Effect size estimation was based on absolute values and log-transformed values. Indexes of HRV in normalized units were not included.

### Data analysis

#### Meta-analytical procedure

Statistical analyses were performed with R, version 3.5.2 (R Core Team, 2018) using the following packages: meta (Schwarzer, 2007), metafor (Viechtbauer, 2010), esc (Lüdecke, 2018) and dplyr (Wickham, Francois, Henry, & Müller, 2015). Hedges' *g* was used as a measure of effect size, since it provides a better estimation of the standardized mean differences in small sample sizes than Cohen's *d* (Borenstein, Hedges, Higgins, & Rothstein, 2009). Quantification of the effect size magnitude for Hedge's *g* is equal to the thresholds defined for Cohen's *d* (Cohen, 1992): small (0.2), medium (0.5) and large (0.8), respectively. Individuals suffering from PTSD represent a heterogeneous population, differing in several aspects such as trauma type or duration of trauma exposure (Brewin, Andrews, & Valentine, 2000). Therefore, random-effects models were chosen, which assume that the true effects in the examined studies are derived from a distribution of true effects (Borenstein et al., 2009).

#### Heterogeneity analysis

Cochran *Q* test was used to examine significance of observed heterogeneity. *I*^2^ index (Higgins & Thompson, 2002) was used to assess degree of heterogeneity. *I*^2^ refers to the amount of variation between studies that is based on true variation in effect size and is interpreted according to benchmarks set by Higgins and Thompsons (2002^2002^): ≈25% low, ≈50% moderate and ≈75% high level of heterogeneity. Prediction intervals (PI) for each effect size were reported, thus allowing to estimate the range of effect sizes of future studies, based on current meta-analytical evidence (IntHout, Ioannidis, Rovers, & Goeman, 2016). Robustness of estimated effects was also examined through outlier detection. In case the confidence interval (CI) of a single study did not overlap with the CI of the aggregated effect size, the study was judged as an outlier. In case of outliers, meta-analysis was rerun without the corresponding outliers. Meta-regressions and subgroup analyses were conducted in case of significant heterogeneity.

#### Moderator variables

A number of variables impact HRV and PTSD, thus calling for the analysis of moderator variables. For example, individuals with PTSD show higher rates of smoking (Kearns et al., 2018) and obesity (Smith, Tyzik, Neylan, & Cohen, 2015) and both these variables influence HRV (Ralevski, Petrakis, & Altemus, 2018; Triggiani et al., 2017). Therefore, these variables were considered as moderators. Gender might be important as well, since women have a higher risk to develop lower HRV after trauma (Insulander & Vallin, 2005; Keary, Hughes, & Palmieri, 2009). Another moderator to consider is age, since HRV declines with aging (Umetani, Singer, McCraty, & Atkionson, 1998). Experience of trauma may not always lead to the development of PTSD (Sahar, Shalev, & Porges, 2001). Hence, type of control group (healthy controls or trauma-exposed without PTSD) as well as type of trauma (interpersonal *v.* non-interpersonal) were investigated. Differences in autonomic arousal might also stem from the presence or absence of dissociative symptoms in PTSD (Seligowski et al., 2019). Therefore, dissociation was included as a moderator. Moreover, PTSD measure (clinical interview *v.* self-report) was examined. PTSD symptom severity might be connected to diminished LF-HRV and HF-HRV in acute distress (Dennis et al., 2016), thus constituting potential moderators. Furthermore, medication use and comorbidities were examined as moderators. Other exploratory moderators were the presence of a stress task, study design (laboratory or ambulatory assessment), sample type, sleep measures, recording position, length of measurement and sampling rate of the ECG. Moderator variables were considered for all examined HRV indices. Moderator analyses were conducted if there were sufficient studies available for subgroup-comparisons and meta-regressions. In order to control for inflation of Type I error due to multiple testing, the Benjamini–Hochberg procedure (Benjamini & Hochberg, 1996) was applied to control for false positive findings among significant moderatos (*p* < 0.05). Moderators that survived this procedure are reported.

#### Publication bias

Publication bias was assessed via different approaches. Visual inspection of funnel plots was used to estimate evidence of publication bias. Asymmetric patterns in the funnel plot indicate publication bias. Regression test by Egger, Smith, Schneider, and Minder (1997) was used to test for funnel plot asymmetry. Regression intercept is supposed to be zero in the absence of publication bias. The trim and fill method (Duval & Tweedie, 2000) was used to adjust for publication bias. Trim and fill describes an iterative approach, where the most extreme effect sizes on the positive side of the funnel plot are trimmed and missing effect sizes are filled into the funnel plot until funnel plot symmetry is reached. The goal is to reach an unbiased estimate of the effect size. P-uniform* is relatively new and constitutes a robust test of publication bias and considers significant and non-significant effect sizes when estimating publication bias (van Aert & van Assen, 2018). P-uniform* is a selection approach model. According to the underlying selection model, probability of publishing statistically significant and non-significant effects sizes is constant, though the probabilities for these two assumptions may differ from each other.

## Results

### Study selection and study characteristics

The studies included in the meta-analysis span two decades (published between 2000 and 2019). Electronic database search on HRV and PTSD revealed 4145 studies. Title and abstract of these studies were screened to evaluate their suitability for full-text review. Non-matching studies (according to predefined inclusion and exclusion criteria, see ‘Methods’ section) and duplicates (the same article found across multiple search engines) were removed, leading to 186 studies suitable for full-text review. In total, 143 studies were excluded due to missing data on HRV. In total, 43 studies were included in the meta-analysis. Four studies came from unpublished datasets. Correlational and categorical data were included in the meta-analyses. Study characteristics of all primary studies included in the meta-analyses are shown in Tables 1–9.

**Table 1.** Description of moderators and inclusion rate

Meta-regression		
Moderator	Description	Inclusion rate (%)^a^
Age	Mean age	83.72
Gender	Percent female	90.68
Length of measurement	Mean recording time (min)	81.40
Obesity	Mean BDI (PTSD)	18.60
Sampling rate	Sampling frequency (Hz)	54.76
Smoking	Percent smoking status % (PTSD)	25.58
PTSD severity	Mean value CAPS (DSM-IV)	23.26
Subgroup analysis		
Moderator	Subgroups	Inclusion rate (%)^a^
Control group	Healthy control / Trauma exposed (no PTSD) / Mixed	88.37
Comorbidities (in PTSD group)	Reported / Not reported	25.58
DSM measure	DSM III / DSM-IV DSM 5	65.12
PTSD measure	Clinical interview / Self report	93.02
Type of participants	Veterans / Others / Mixed	72.09
Medication	Reported / Not reported	60.47
Presence of stress task	Present / Not present	93.02
Study design	Laboratory / Ambulatory assessment	90.70
Type of trauma	Interpersonal / Non-interpersonal / Mixed	76.74
Recording position	Supine / Sitting	51.16
Sleep measures	Yes / No	11.63
Dissociative symptoms	Reported / Not reported	6.98

a Inclusion rate = percent of studies reporting information on the moderator.

**Table 2.** Characteristics of studies included in meta-analysis 1 (RMSSD)

Study	*N*	Hedges’ *g*	weight%	Cl. lb	Cl. ub
Agorastos et al. (2013)	15	−1.02	3.3	−2.10	0.07
Chang, Shin, Kim, and Chung (2016)	23	−1.00	4.3	−1.87	−0.13
DePierro (2015, unpublished data)	65	−0.20	6.8	−0.63	0.28
Hauschildt, Peters, Moritz, and Jelinek (2011)	52	−0.49	6.3	−1.04	0.06
Lakusic et al. (2007)	68	−0.59	6.8	−1.08	−0.11
Lee and Theus (2012)	125	−0.56	7.5	−0.95	−0.17
Liu, Bing, Yong, Xie, and Sen (2019)	152	−0.17	8.0	−0.49	0.14
Meyer et al. (2016)	41	−1.00	5.6	−1.65	−0.34
Minassian et al. (2014)	2235	0.21	8.8	0.02	0.39
Moon, Lee, Kim, and Hwang (2013)	61	−0.64	6.6	−1.16	−0.12
Norte et al. (2013)	35	−1.08	5.2	−1.80	−0.37
Park et al. (2017)	141	−0.57	7.9	−0.91	−0.24
Shaikh al arab et al. (2012)	21	−0.84	4.2	−1.73	0.06
Song et al. (2011)	24	0.42	4.6	−0.40	1.24
Spiller et al. (2019)	81	0.08	6.8	−0.40	0.56
Thome et al. (2017)	98	0.44	7.4	0.04	0.85

**Table 3.** Characteristics of studies included in meta-analysis 2 (SDNN)

Study	*N*	Hedges’ *g*	weight%	Cl. lb	Cl. ub
Agorastos et al. (2013)	15	−0.57	4.7	−1.60	0.47
Brady et al. (2015)	114	−0.28	7.0	−0.65	0.09
Chang et al. (2016)	23	−0.98	5.3	−1.85	−0.11
Dennis et al. (2014)	227	−0.32	7.2	−0.58	−0.06
DePierro (2015, unpublished data)	65	−0.17	6.6	−0.66	0.32
Lakusic et al. (2007)	68	−0.24	6.7	−0.72	0.24
Lee and Theus (2012)	125	−0.61	6.9	−1.00	−0.22
Liu et al. (2019)	152	−2.64	6.8	−3.07	−2.20
Meyer et al. (2016)	41	−0.94	6.1	−1.59	−0.29
Minassian et al. (2014)	2235	0.16	7.4	−0.02	0.34
Moon et al. (2013)	61	−0.63	6.5	−1.15	−0.12
Park et al. (2017)	141	−0.59	7.1	−0.93	−0.25
Shaikh al arab et al. (2012)	21	−0.84	5.5	−1.74	0.05
Song et al. (2011)	24	0.33	5.5	−0.48	1.15
Tan et al. (2009)	28	−0.40	5.7	−1.15	0.36
Tan, Dao, Farmer, Sutherland, and Gevirtz (2011)	30	−1.84	5.2	−2.74	−0.94

**Table 4.** Characteristics of studies included in meta-analysis 3 (HF-HRV)

Study	*N*	Hedges’ *g*	weight%	Cl. lb	Cl. ub
Blechert, Michael, Grossman, Lajtman, and Wilhelm (2007)	55	−0.74	3.2	−1.30	−0.19
Bertram et al. (2014)	46	0.12	3.0	−0.46	0.70
Brady et al. (2015)	114	−0.14	5.2	−0.51	0.23
Chang et al. (2013)	224	−0.61	5.0	−0.99	−0.24
Cohen et al. (2000)	39	−0.28	2.5	−0.93	0.38
Dennis et al. (2014)	227	−0.41	6.9	−0.67	−0.14
DePierro (2015, unpublished data)	65	−0.33	3.7	−0.82	0.15
Hauschildt et al. (2011)	52	−0.49	3.2	−1.04	0.06
Jovanovic et al. (2009)	78	−0.76	4.0	−1.23	−0.30
Keary et al. (2009)	40	0.43	2.6	−0.19	1.06
Lakusic et al. (2007)	68	−0.06	3.9	−0.53	0.42
Lee et al. (2018)	102	−0.43	4.8	−0.82	−0.03
Liverant (2016, unpublished data)	79	0.10	1.5	−0.78	0.98
Meyer et al. (2016)	41	−0.14	2.7	−0.75	0.48
Minassian et al. (2014)	2235	−0.23	8.3	−0.42	−0.05
Moon et al. (2013)	61	−0.14	3.6	−0.65	0.34
Park et al. (2017)	141	−0.71	5.6	−1.05	−0.33
Shah et al. (2013)	416	−0.41	5.2	−0.78	−0.04
Slewa-Younan et al. (2012)	35	−0.16	2.2	−0.85	0.54
Song et al. (2011)	24	0.14	1.8	−0.67	0.95
Thome et al. (2017)	98	−0.38	4.7	−0.79	0.02
Tucker, Pfefferbaum, Jeon-Slaughter, Khan, and Garton (2012)	45	0.19	2.5	−0.46	0.83
van Male (2000, unpublished data)	30	0.20	0.5	−1.52	1.92
Wahbeh and Oken (2013a)	30	0.34	2.1	−0.38	1.06
Wahbeh and Oken (2013b)	81	0.00	4.1	−0.45	0.45
Wisco (2015, unpublished data)	10	−0.46	0.8	−1.77	0.85
Woodward, Kaloupek, Schaer, Martinez, and Eliez (2008)	77	0.20	4.2	−0.25	0.64
Woodward et al. (2009)	35	0.45	2.3	−0.25	1.14

**Table 5.** Characteristics of studies included in meta-analysis 4 (LF-HRV)

Study	*N*	Hedges’ *g*	weight%	Cl. lb	Cl. ub
Brady et al. (2015)	114	−0.47	7.0	−0.84	−0.10
Chang et al. (2013)	224	−0.51	6.9	−0.89	−0.14
Cohen et al. (2000)	39	−0.04	2.9	−0.69	0.61
Dennis et al. (2014)	227	−0.40	10.6	−0.66	−0.13
DePierro (2015, unpublished data)	65	−0.14	4.7	−0.63	0.34
Hauschildt et al. (2011)	52	−0.64	3.8	−1.20	−0.09
Keary et al. (2009)	40	0.00	3.2	−0.62	0.62
Lakusic et al. (2007)	68	0.07	4.9	−0.41	0.54
Meyer et al. (2016)	41	−0.13	3.2	−0.75	0.49
Minassian et al. (2014)	2235	−0.24	14.5	−0.43	−0.06
Moon et al. (2013)	61	−0.11	4.4	−0.62	0.39
Park et al. (2017)	141	−0.15	8.2	−0.48	0.18
Shah et al. (2013)	416	−0.67	7.1	−1.04	−0.30
Slewa-Younan et al. (2012)	35	−0.17	2.6	−0.87	0.53
Song et al. (2011)	24	0.59	1.9	−0.24	1.42
Thome et al. (2017)	98	−0.57	6.1	−0.98	−0.16
Tucker et al. (2012)	45	0.22	2.9	−0.42	0.87
Wahbeh and Oken (2013b)	81	−0.06	5.2	−0.52	0.39

**Table 6.** Characteristics of studies included in meta-analysis 5 (LF/HF)

Study	*N*	Hedges’ *g*	weight%	Cl. lb	Cl. ub
Agorastos et al. (2013)	15	1.14	5.1	0.04	2.24
Chang et al. (2013)	224	0.14	8.7	−0.23	0.51
Clausen, Aupperle, Sisante, Wilson, and Billinger (2016)	24	0.87	6.3	0.03	1.71
Kobayashi, Lavela, and Mellman (2014)	37	0.49	7.3	−0.17	1.15
Lakusic et al. (2007)	68	1.29	8.0	0.77	1.81
Lee et al. (2018)	102	0.48	8.6	0.08	0.87
Mellman, Knorr, Pigeon, Leiter, and Akay (2004)	19	0.62	5.9	−0.31	1.54
Minassian et al. (2014)	2235	0.08	9.4	−0.11	0.26
Mitani, Fujita, Sakamoto, and Shirakawa (2006)	22	1.87	5.4	0.86	2.89
Moon et al. (2013)	61	0.50	8.0	−0.02	1.01
Slewa-Younan et al. (2012)	35	0.34	7.0	−0.36	1.05
Song et al. (2011)	24	0.55	6.4	−0.28	1.38
Tucker et al. (2012)	45	3.50	5.6	2.52	4.48
Wahbeh and Oken (2013b)	81	−0.25	8.3	−0.71	0.20

**Table 7.** Characteristics of studies included in meta-analysis 6 (HR)

Study	*N*	Hedges’ *g*	weight%	Cl. lb	Cl. ub
Agorastos et al. (2013)	15	1.20	3.9	0.08	2.30
Bertram et al. (2014)	61	0.63	6.2	0.11	1.15
Cohen et al. (2000)	39	2.44	4.8	1.58	3.29
DePierro (2015, unpublished data)	65	0.63	6.2	0.63	1.13
Hauschildt et al. (2011)	44	0.35	5.8	−0.25	0.95
Jovanovic et al. (2009)	78	0.75	6.4	0.28	1.21
Keary et al. (2009)	40	0.26	5.8	−0.36	0.88
Liu et al. (2019)	152	2.01	6.6	1.62	2.40
Mellman et al. (2004)	19	0.32	4.7	−0.58	1.23
Meyer et al. (2016)	41	0.23	5.8	−0.38	0.85
Norte et al. (2013)	35	0.99	5.4	0.28	1.70
Park et al. (2017)	141	0.50	6.8	0.17	0.84
Shaikh al arab et al. (2012)	21	1.52	4.4	0.54	2.50
Slewa-Younan et al. (2012)	35	1.77	5.0	0.95	2.59
Song et al. (2011)	24	0.22	5.0	−0.59	1.04
Wahbeh and Oken (2013a)	30	0.12	5.4	−0.60	0.83
Woodward et al. (2008)	77	0.06	6.4	−0.39	0.51
Woodward et al. (2009)	35	0.61	5.5	−0.09	1.31

**Table 8.** Characteristics of studies included in meta-analysis 7 (HR stress activity)

Study	*N*	Hedges’ *g*	weight%	Cl. lb	Cl. ub
Cohen et al. (2000)	39	0.76	18.9	0.08	1.43
Jovanovic et al. (2009)	78	0.42	42.0	−0.04	0.87
Keary et al. (2009)	40	0.32	22.2	−0.30	0.95
Wahbeh and Oken (2013a)	30	0.10	16.9	−0.62	0.81

**Table 9.** Characteristics of studies included in meta-analysis 8 (HF-HRV stress activity)

Study	*N*	Hedges’ *g*	weight%	Cl. lb	Cl. ub
Cohen et al. (2000)	39	−0.10	10.1	−0.76	0.55
Dennis et al. (2016)	219	−0.06	19.7	−0.32	0.21
Jovanovic et al. (2009)	78	−0.77	14.2	−1.23	−0.30
Kamkwalala et al. (2012)	54	0.51	11.5	−0.07	1.09
Kamkwalala et al. (2012)	87	−0.40	14.7	−0.85	0.04
Keary et al. (2009)	40	−0.62	10.5	−1.26	0.01
Tucker et al. (2012)	45	−0.03	10.3	−0.67	0.62
Wahbeh and Oken (2013a)	30	−0.41	9.0	−1.14	0.31

Average age of the examined samples was 38.81 years. Thirty studies were laboratory studies, nine studies were ambulatory studies and in four studies information about study design was not available. Thirty-three studies reported the type of trauma, with interpersonal trauma being the most frequently reported trauma type. In the majority of studies, PTSD diagnosis was based on the Diagnostic and Statistical Manual of Mental Disorders, 4th edition (DSM-IV, American Psychiatric Association, 1994). One study used the Minnesota Multiphasic Personality Inventory (MMPI-PTSD; Keane, Malloy, and Fairbank, 1984), one study used the Chinese Version of the Modified Schedule of Affective disorder and Schizophrenia-Lifetime (SADSL, Endicott & Spitzer, 1978) and one study used the International Statistical Classification of Diseases and Related Health Problems (ICD-9). One study utilized the Impact of Event Scale-revised (IES-R, Asukai et al., 2002) for PTSD diagnosis, using a cut-off score of 18 points. PTSD symptom severity was analyzed through the symptom severity score of the Clinical Administrated PTSD scale for DSM-IV (CAPS; Blake et al., 1998). Symptom severity scores ranged from 42.0 to 74.59 points, indicating moderate to severe PTSD symptomatology (Weathers, Keane, & Davidson, 2001). Duration of time passed since the last traumatic event ranged from 2 months to 27.8 years. Fourteen studies reported recording HRV in a sitting position and seven studies in a supine position. Shortest measurement length of HRV was half a minute. Sampling rate ranged from 200 to 1440 Hz. Reported diagnosed psychiatric comorbidities were reported in 11 studies, comprising the following classes of mental disorders: depressive and anxiety disorders, eating disorders, personality disorders, substance-related and addictive disorders and somatic symptom disorders.

### Effect sizes at baseline

#### Meta-analysis 1 – time-domain: root mean square of successive heart beat (RMSSD)

Sixteen studies were included in the meta-analysis (*n* = 3237). RMSSD was reduced in individuals with PTSD as compared to controls [Hedges' *g* = −0.38 (95% CI −0.62 to −0.13; PI = −1.30 to 0.55), *p* = 0.003], indicating a small effect size. Two outliers were identified. Exclusion of the outliers led to a slight increase in the effect size [Hedges' *g* = −0.49 (95% CI −0.68 to −0.29; PI = −1.05 to 0.08), *p* < 0.001]. Significant *Q* statistic (*Q* = 64.91, *p* < 0.001) indicated heterogeneity. Heterogeneity of the effect size was high (*I*^2^ = 76.9%). Evidence for publication bias was revealed through Eggers regression test (*p* = 0.01). Trim and fill test indicated five missing studies. Adding the missing studies to the right part of the funnel plot changed the effect size substantially [Hedges' *g* = −0.16 (95% CI −0.40 to 0.08; PI = −1.17 to 0.85), *p* = 0.19], leading to a non-significant effect. Moderator analysis did not find significant moderators.

#### Meta-analysis 2 – time-domain: standard deviation of NN intervals (SDNN)

Sixteen studies were included in the meta-analysis (*n* = 3370). The aggregated effect size for SDNN was medium [Hedges' *g* = −0.64 (95% CI −1.01 to −0.27; PI = −2.17 to 0.88), *p* < 0.001], indicating that individuals with PTSD had smaller SDNN as compared to controls. Two outliers were found. Exclusion of the outliers lead to a decrease in the effect size [Hedges' *g* = −0.50 (95% CI −0.69 to −0.31; PI = −1.02 to 0.02), *p* < 0.001]. High levels of heterogeneity were detected (*I*^2^ = 90.6%). Tests for publication bias did not indicate evidence for publication bias. Moderator analysis revealed sampling rate as a possible moderator variable (*p* = 0.01). Sampling rate ranged from 250 to 1024 Hz. A higher sampling rate was associated with a smaller effect size.

#### Meta-analysis 3 – frequency-domain: high-frequency HRV (HF-HRV)

Twenty-eight studies were included in the meta-analysis (*n* = 4548). Group differences were also evident in the HF-HRV parameter [Hedges' *g* = −0.23 (95% CI −0.34 to −0.11; PI = −0.65 to 0.20), *p* < 0.001]. The aggregated effect size was small. No outliers were detected and heterogeneity was low (*I*^2^ = 44.6%). There was no evidence for publication bias. Significant moderators were not detected.

#### Meta-analysis 4 – frequency-domain: low-frequency HRV (LF-HRV)

Eighteen studies were included in the meta-analysis (*n* = 4006). LF-HRV was smaller in individuals with PTSD as compared to controls. The aggregated effect size was small [Hedges' *g* = −0.27 (95% CI −0.39 to −0.14; PI = −0.57 to 0.03), *p* ≤ 0.001]. No outliers were observed. The level of heterogeneity was low (*I*^2^ = 27.6%). Evidence for publication bias was not present.

#### Meta-analysis 5 – frequency-domain: low-frequency/high-frequency ratio (LF/HF)

Fourteen studies were included in the meta-analysis (*n* = 2992). A medium effect size was detected for LF/HF ratio [Hedges' *g* = 0.72 (95% CI 0.36 to 1.08; PI = −0.69 to 2.08), *p* < 0.001]. Three outliers were detected. Exclusion of this outlier led to a decrease in the effect size [Hedges' *g* = 0.66 (95% CI 0.38–0.94; PI = −0.14 to 1.47), *p* < 0.001]. The level of heterogeneity was high (*I*^2^ = 84.2%). Egger's regression test (*p* < 0.001) revealed evidence of publication bias. Trim and fill test indicated six missing studies. Adding the missing studies to the left part of the funnel plot changed the effect size substantially [Hedges' *g* = 0.22 (95% CI −0.17 to 0.60; PI = −1.50 to 1.94), *p* = 0.27], leading to a non-significant effect. Moderator analyses did not lead to a reduction in heterogeneity.

#### Meta-analysis 6 – heart rate (HR)

Eighteen studies were included in the meta-analysis (*n* = 952). HR was significantly higher in individuals with PTSD compared to controls [Hedges' *g* = 0.78 (95% CI 0.46 to 1.11; PI = −0.56 to 2.13), *p* < 0.001]. Two outliers were detected. Exclusion of the outliers led to a slight decrease in the effect size [Hedges' *g* = 0.56 (95% CI 0.36 to 0.76; PI = −0.01 to 1.14), *p* < 0.001]. No evidence for publication bias was found. High level of heterogeneity was observed (*I*^2^ = 80.8%). Significant moderators were not detected.

### Effect sizes during stress

#### Meta-analyses 7 and 8 – HR and HRV stress activity

Four studies were included in the analysis of HR during stress (*n* = 187). Individuals with PTSD evidenced higher HR during a stress task as compared to controls [Hedges' *g* = 0.41 (95% CI 0.11–0.70; PI = −0.24 to 1.05), *p* < 0.001]. No heterogeneity was observed (*I*^2^ = 0.0%). Eight studies were included in the analysis of HF-HRV (*n* = 592). Individuals with PTSD tended to show lower HF-HRV during the stress task compared to controls [Hedges' *g* = −0.24 (95% CI −0.50 to −0.03; PI = −1.00 to 0.54), *p* = 0.089]; however, effect size did not reach significance. Moderate heterogeneity was observed (*I*^2^ = 55.3%). Due to the low numbers of included studies in the meta-analyses on stress activity, no moderator analyses were carried out.

## Discussion

The aim of this study was to examine differences in HRV parameters between individuals with PTSD and healthy controls at rest and during stress. Results indicate that individuals with PTSD have lower HRV, as compared to healthy controls, both at rest and during stress. Small negative effect sizes in RMSSD, HF-HRV and LF-HRV suggest reduced parasympathetic (i.e., vagal) activity in individuals with PTSD, as compared to controls. The moderate negative effect in SDNN highlights diminished total variability in PTSD. The positive effect size in the LF/HF ratio possibly suggests changes in sympatho-vagal balance in PTSD, and increased HR in PTSD at baseline and during stress may indicate higher SNS activity. Results suggest that changes in the ANS in individuals with PTSD are not restricted to pure vagally-mediated HRV parameters, but may rather indicate a general ANS dysregulation.

Importantly, the performed meta-analyses show predominantly high levels of heterogeneity, which may be due to relatively small sample sizes in the primary studies (median *N* = 46). Moderator variables such as physical health and physical activity, as well as physical and psychiatric comorbidities might also contribute to high heterogeneity. Moderator analysis did not reveal significant moderatos, with one exception being SDNN, where a higher sampling rate seems to be accompanied by smaller effect sizes. It is particularly surprising that moderator analyses on gender did not reveal significant results, since there is evidence for sex differences in PTSD (Tolin & Foa, 2006) and HRV (Koenig & Thayer, 2016). One possible reason for not being able to reveal significant moderators might be the relatively limited number of studies that provided sufficient information on moderators, hence questioning the robustness of the moderator analyses. Publication bias was evident in two meta-analyses, namely LF/HF ratio and RMSSD. Of note, a homogeneous pattern of physiological aberrations in PTSD might be questioned, since individuals suffering from PTSD show diverse symptoms, an example being some individuals who do not develop alterations in arousal.

Our findings generally support the results of the previous meta-analysis on PTSD and HRV by Nagpal et al. (2013) and extend the meta-analysis performed by Campbell et al. (2019). Although the direction of effects is identical in all performed meta-analyses, they diverge in magnitude. Beside our study, only Nagpal et al. (2013) meta-analyzed differences in LF-HRV in individuals with PTSD and controls. Due to exclusion of unpublished data and a smaller number of studies included, they reported a comparably large effect for LF-HRV (Hedges' *g* = −1.72), which might overestimate the true effect. In the current meta-analysis, differences in LF-HRV between individuals with PTSD and controls were indicated by a small effect size. Publication bias was not evident, suggesting a more robust effect size estimation. LF-HRV has been related to different ANS mechanisms, reflecting a mixture of parasympathetic and sympathetic activity in long-term ambulatory studies, and primarily parasympathetic activity in resting state assessments in the laboratory (e.g. Shaffer, McCraty, and Zerr, 2014). Hence, the findings suggest a general reduction of HRV in PTSD individuals as compared to controls.

One remaining question is, if lower HRV constitutes a risk factor for developing PTSD (vulnerability marker), or if lower HRV is a factor that develops during the course of PTSD (scar marker). Rombold-Bruehl et al. (2019) revealed that low HRV might impose risk for the frequency and recovery from intrusive memories, thus suggesting enhanced vulnerability. To examine the role of HRV as a scar marker, longitudinal studies are needed, which examine changes in HRV before a traumatic event, and within the development of PTSD. So far, mainly cross-sectional studies examined the relationship between HRV and PTSD. Hence, no causal relationship can be derived from the calculated effect sizes. Certainly, more research is needed to elucidate the role of HRV in the development of PTSD.

The findings of this meta-analysis are limited by the shortcomings of the primary studies included, and the general availability of studies within this field. First, due to high levels of heterogeneity interpretations of effects should be made with caution. Publication bias was evident in the meta-analyses on RMSSD and LF/HF ratio, thus suggesting rather fragile effects. Second, lower HRV in PTSD might be related to specific features of PTSD. Dennis et al. (2014) showed that sleep disturbances in individuals with PTSD mediated the association between PTSD and HRV. Differences in PTSD symptom expressions could not be examined in the current meta-analyses. Third, due to missing data in primary studies, data on stress reactivity (relative to baseline), as well as recovery, could not be included. Impaired recovery seems to be a main aspect of PTSD symptoms (Pole, 2007), which calls for further research.

In conclusion, the current meta-analysis supports previous reviews suggesting that PTSD is associated with lower HRV, thus highlighting the need for a stronger focus on examining HRV changes within the development and course of PTSD. The results highlight that not a single HRV parameter is particularly indicative for PTSD. Rather, changes in HRV occurred in vagally-mediated HRV parameters, as well as in more complex measures, thus indicating a general pattern of ANS dysregulation. Of note, alterations in ANS functioning in individuals with PTSD seem to be evident during both rest and stress. High levels of heterogeneity indicate substantial variance between studies included in the analyses. The majority of the examined moderator variables could not explain this heterogeneity. Therefore, the focus on identifying moderator variables that influence the relationship between PTSD and low HRV seems crucial for gaining a better understanding of the psychophysiological connections between HRV and PTSD.

## Supplementary material

For supplementary material accompanying this paper visit https://doi.org/10.1017/S003329172000207X. click here to view supplementary material

## References

[ref1] *Agorastos, A., Boel, J. A., Heppner, P. S., Hager, T., Moeller-Bertram, T., Haji, U., … Stiedl, O. (2013). Diminished vagal activity and blunted diurnal variation of heart rate dynamics in posttraumatic stress disorder. Stress (Amsterdam, Netherlands), 16(3), 300–310. doi:10.3109/10253890.2012.751369. 23167763

[ref2] American Psychiatric Association (1994). Diagnostic and statistical manual of mental disorders (4th ed., Text Revision). Washington DC: Author.

[ref3] American Psychiatric Association (2013). Diagnostic and statistical manual of mental disorders (5th ed.) Arlington VA: Author.

[ref4] Asukai, N., Kato, H., Kawamura, N., Kim, Y., Yamamoto, K., Kishimoto, J., … Nishizono-Maher, A. (2002). Reliability and validity of the Japanese-language version of the impact of event scale-revised (Ies-RJ): Four studies of different traumatic events. The Journal of Nervous and Mental Disease, 190(3), 175–182. Retrieved from https://journals.lww.com/jonmd/Abstract/2002/03000/RELIABILIGY_AND_VALIDITY_OF_THE_JAPANESE_LANGUAGE.6.aspx. 11923652 10.1097/00005053-200203000-00006

[ref5] Benjamini, Y., & Hochberg, Y. (1996). Controlling the false discovery rate: A practical and powerful approach to multiple testing. Journal of the Royal Statistical Society B, 57, 289–300.

[ref6] *Bertram, F., Jamison, A. L., Slightam, C., Kim, S., Roth, H. L., & Roth, W. T. (2014). Autonomic arousal during actigraphically estimated waking and sleep in male veterans with PTSD. Journal of Traumatic Stress, 27(5), 610–617. doi:10.1002/jts.21947. 25322890

[ref7] Blake, D. D., Weathers, F. W., Nagy, L. M., Kaloupek, D. G., Charney, D. S., & Keane, T. M. (1998). Clinician-administered PTSD scale for DSM-IV. Boston: National Center for Posttraumatic Stress Disorder.

[ref8] *Blechert, J., Michael, T., Grossman, P., Lajtman, M., & Wilhelm, F. H. (2007). Autonomic and respiratory characteristics of posttraumatic stress disorder and panic disorder. Psychosomatic Medicine, 69(9), 935–943. doi:10.1097/PSY.0b013e31815a8f6b. 17991823

[ref9] Borenstein, M., Hedges, L. V., Higgins, J. P., & Rothstein, H. R. (2009). Introduction to meta-analysis. Chichester: John Wiley & Sons.

[ref10] *Brady, R. E., Constans, J. I., Marx, B. P., Spira, J. L., Gevirtz, R., Kimbrell, T. A., … Pyne, J. M. (2015). Effect of symptom over-reporting on heart rate variability in veterans with posttraumatic stress disorder. Journal of Trauma & Dissociation, 16(5), 551–562. doi:10.1080/15299732.2015.1021505. 26011249

[ref11] Brewin, C. R., Andrews, B., & Valentine, J. D. (2000). Meta-analysis of risk factors for posttraumatic stress disorder in trauma-exposed adults. Journal of Consulting and Clinical Psychology, 68(5), 748. doi:10.1037/0022-006X.68.5.748. 11068961

[ref12] Camm, A. J., Malik, M., Bigger, J. T., Breithardt, G., Cerutti, S., Cohen, R. J., … Lombardi, F. (1996). Heart rate variability: Standards of measurement, physiological interpretation and clinical use (Task Force of the European Society of Cardiology and the North American Society of Pacing and Electrophysiology). Circulation, 93, 1043–1065. doi:10.1161/01.CIR.93.5.1043. 8598068

[ref13] Campbell, A. A., Wisco, B. E., Silvia, P. J., & Gay, N. G. (2019). Resting respiratory sinus arrhythmia and posttraumatic stress disorder: A meta-analysis. Biological Psychology, 144, 125–135. doi:10.1016/j.biopsycho.2019.02.005. 30779926

[ref14] *Chang, H. A., Chang, C. C., Tzeng, N. S., Kuo, T. B., Lu, R. B., & Huang, S. Y. (2013). Decreased cardiac vagal control in drug-naïve patients with posttraumatic stress disorder. Psychiatry Investigation, 10(2), 121. doi:10.4306/pi.2013.10.2.121. 23798959 PMC3687045

[ref15] *Chang, H. Y., Shin, K. M., Kim, N. H., & Chung, Y. K. (2016). PS263. Association between heart rate variability (HRV) and posttraumatic stress symptoms in female victims of sexual violence. International Journal of Neuropsychopharmacology, 19(Suppl. 1), 95. doi:10.1093/ijnp/pyw043.263.

[ref16] *Clausen, A. N., Aupperle, R. L., Sisante, J. F. V., Wilson, D. R., & Billinger, S. A. (2016). Pilot investigation of PTSD, autonomic reactivity, and cardiovascular health in physically healthy combat veterans. PLoS ONE, 11(9), e0162547. doi:10.1371/journal.pone.0162547. 27607181 PMC5015867

[ref17] Cohen, J. (1992). A power primer. Psychological Bulletin, 112, 155–159. Retrieved from https://psycnet.apa.org/buy/1992-37683-001. 19565683 10.1037//0033-2909.112.1.155

[ref18] *Cohen, H., Benjamin, J., Geva, A. B., Matar, M. A., Kaplan, Z., & Kotler, M. (2000). Autonomic dysregulation in panic disorder and in post-traumatic stress disorder: Application of power spectrum analysis of heart rate variability at rest and in response to recollection of trauma or panic attacks. Psychiatry Research, 96(1), 1–13. doi:10.1016/S0165-1781(00)00195-5. 10980322

[ref19] de Godoy, M. F. (2016). Nonlinear analysis of heart rate variability: A comprehensive review. Journal of Cardiology and Therapy, 3(3), 528–533. Retrieved from http://ghrnet.org/index.php/jct/article/view/1724.

[ref20] *Dennis, P. A., Dedert, E. A., Van Voorhees, E. E., Watkins, L. L., Hayano, J., Calhoun, P. S., … Beckham, J. C. (2016). Examining the crux of autonomic dysfunction in PTSD: Whether chronic or situational distress underlies elevated heart rate and attenuated heart-rate variability. Psychosomatic Medicine, 78(7), 805. doi:10.1097/PSY0000000000000326. 27057817 PMC5003742

[ref21] *Dennis, P. A., Watkins, L., Calhoun, P. S., Oddone, A., Sherwood, A., Dennis, M. F., … Beckham, J. C. (2014). Posttraumatic stress, heart-rate variability, and the mediating role of behavioral health risks. Psychosomatic Medicine, 76(8), 629. doi:10.1097/PSY.0000000000000110. 25264973 PMC4197067

[ref22] Duval, S., & Tweedie, R. (2000). Trim and fill: A simple funnel-plot-based method of testing and adjusting for publication bias in meta-analysis. Biometrics, 56(2), 455–463. doi:10.1111/j.0006-341C.2000.00455.x. 10877304

[ref23] Egger, M., Smith, G. D., Schneider, M., & Minder, C. (1997). Bias in meta-analysis detected by a simple, graphical test. BMJ, 315(7109), 629–634. doi:10.1136/bmj.315.7109.629. 9310563 PMC2127453

[ref24] Elliott, K., & Lawrenson, G. (Eds.). (2009). Development of the autonomic nervous system. Chichester: John Wiley & Sons.

[ref25] Endicott, J., & Spitzer, R. L. (1978). A diagnostic interview: The schedule for affective disorders and schizophrenia. Archives of General Psychiatry, 35(7), 837–844. 678037 10.1001/archpsyc.1978.01770310043002

[ref26] Goldstein, D. S., Bentho, O., Park, M. Y., & Sharabi, Y. (2011). Low-frequency power of heart rate variability is not a measure of cardiac sympathetic tone but may be a measure of modulation of cardiac autonomic outflows by baroreflexes. Experimental Physiology, 96(12), 1255–1261. doi:10.1113/expphysiol.2010.056259. 21890520 PMC3224799

[ref27] *Hauschildt, M., Peters, M. J., Moritz, S., & Jelinek, L. (2011). Heart rate variability in response to affective scenes in posttraumatic stress disorder. Biological Psychology, 88(2-3), 215–222. doi:10.1016/j.biopsycho.2011.08.004. 21856373

[ref28] Higgins, J. P., & Green, S. (Eds.). (2011). Cochrane handbook for systematic reviews of interventions. Chichester: John Wiley & Sons.

[ref29] Higgins, J. P., & Thompson, S. G. (2002). Quantifying heterogeneity in a meta-analysis. Statistics in Medicine, 21(11), 1539–1558. doi:10.1002/sim.1186. 12111919

[ref30] Hillebrand, S., Gast, K. B., de Mutsert, R., Swenne, C. A., Jukema, J. W., Middeldorp, S., … Dekkers, O. M. (2013). Heart rate variability and first cardiovascular event in populations without known cardiovascular disease: Meta-analysis and dose–response meta-regression. Europace, 15(5), 742–749. doi:10.1093/europace/eus341. 23370966

[ref31] Insulander, P., & Vallin, H. (2005). Gender differences in electrophysiologic effects of mental stress and autonomic tone inhibition: A study in healthy individuals. Journal of Cardiovascular Electrophysiology, 16(1), 59–63. doi:10.1046/j.1540-8167.2005.04177.x. 15673389

[ref32] IntHout, J., Ioannidis, J. P., Rovers, M. M., & Goeman, J. J. (2016). Plea for routinely presenting prediction intervals in meta-analysis. BMJ Open, 6(7), e010247. doi:10.1136/bmjopen-2015-010247. PMC4947751 27406637

[ref33] Jänig, W. (2008). Integrative action of the autonomic nervous system: Neurobiology of homeostasis. Cambridge: Cambridge University Press.

[ref34] Johnson, B. K. (2018). Physiology of the autonomic nervous system In E. Farag, M. Argalious, J.E. Tetzlaff & D. Sharma (Eds.), Basic sciences in anesthesia (pp. 355–364). Cham: Springer. doi:10.1007/978-3-319-62067-1_19.

[ref35] *Jovanovic, T., Norrholm, S. D., Sakoman, A. J., Esterajher, S., & Kozarić-Kovačić, D. (2009). Altered resting psychophysiology and startle response in Croatian combat veterans with PTSD. International Journal of Psychophysiology, 71(3), 264–268. doi:10.1016/j.ijpsycho.2008.10.007. 19013485 PMC3749920

[ref36] *Kamkwalala, A., Norrholm, S. D., Poole, J. M., Brown, A., Donley, S., Duncan, E., … Jovanovic, T. (2012). Dark-enhanced startle responses and heart rate variability in a traumatized civilian sample: Putative sex-specific correlates of posttraumatic stress disorder. Psychosomatic Medicine, 74(2), 153. doi:10.1097/PSY.0b013e318240803a. 22286850 PMC3674026

[ref37] Keane, T. M., Malloy, P. F., & Fairbank, J. A. (1984). Empirical development of an MMPI subscale for the assessment of combat-related posttraumatic stress disorder. Journal of Consulting and Clinical Psychology, 52(5), 888. doi:10.1037/0022-006X.52.5.888. 6501674

[ref38] Kearns, N. T., Carl, E., Stein, A. T., Vujanovic, A. A., Zvolensky, M. J., Smits, J. A., & Powers, M. B. (2018). Posttraumatic stress disorder and cigarette smoking: A systematic review. Depression and Anxiety, 35(11), 1056–1072. doi:10.1002/da.22828. 30192425

[ref39] *Keary, T. A., Hughes, J. W., & Palmieri, P. A. (2009). Women with posttraumatic stress disorder have larger decreases in heart rate variability during stress tasks. International Journal of Psychophysiology, 73(3), 257–264. doi:10.1016/j.ijpsycho.2009.04.003. 19374925

[ref40] *Kobayashi, I., Lavela, J., & Mellman, T. A. (2014). Nocturnal autonomic balance and sleep in PTSD and resilience. Journal of Traumatic Stress, 27(6), 712–716. doi:10.1002/jts.21973. 25403523

[ref41] Koenig, J., & Thayer, J. F. (2016). Sex differences in healthy human heart rate variability: A meta-analysis. Neuroscience & Biobehavioral Reviews, 64, 288–310. doi:10.1016/j.neubiorev.2016.03.007. 26964804

[ref42] *Lakusic, N., Fuckar, K., Mahovic, D., Cerovec, D., Majsec, M., & Stancin, N. (2007). Characteristics of heart rate variability in war veterans with post-traumatic stress disorder after myocardial infarction. Military Medicine, 172(11), 1190-1193. doi:10.7205/MILMED.172.11.1190. 18062395

[ref43] *Lee, S. M., Han, H., Jang, K. I., Huh, S., Huh, H. J., Joo, J. Y., & Chae, J. H. (2018). Heart rate variability associated with posttraumatic stress disorder in victims’ families of Sewol ferry disaster. Psychiatry Research, 259, 277–282. doi:10.1016/j.psychres.2017.08.062. 29091829

[ref44] *Lee, E. A. D., & Theus, S. A. (2012). Lower heart rate variability associated with military sexual trauma rape and posttraumatic stress disorder. Biological Research for Nursing, 14(4), 412–418. doi:10.1177/1099800412454453. 22899708

[ref45] *Liu, C. X., Bing, B. I. N., Yong, M. A., Xie, Z. Y., & Sen, L. I. (2019). Effect of post-traumatic stress disorder on human blood pressure, heart rate, and heart rate variability. Medical Journal of Chinese People's Liberation Army, 44(2), 162–165. doi:10.11855/j.issn.0577-7402.2019.02.12.

[ref46] Lüdecke, D. (2018). esc: Effect Size Computation for Meta Analysis. R package version 0.4.1.

[ref47] McCraty, R., & Shaffer, F. (2015). Heart rate variability: New perspectives on physiological mechanisms, assessment of self-regulatory capacity, and health risk. Global Advances in Health and Medicine, 4(1), 46–61. doi:10.7453/gahmj.2014.073. PMC4311559 25694852

[ref48] *Mellman, T. A., Knorr, B. R., Pigeon, W. R., Leiter, J. C., & Akay, M. (2004). Heart rate variability during sleep and the early development of posttraumatic stress disorder. Biological Psychiatry, 55(9), 953–956. doi:10.1016/j.biopsych.2003.12.018. 15110740

[ref49] *Meyer, P. W., Mueller, L. E., Zastrow, A., Schmidinger, I., Bohus, M., Herpertz, S. C., & Bertsch, K. (2016). Heart rate variability in patients with post-traumatic stress disorder or borderline personality disorder: Relationship to early life maltreatment. Journal of Neural Transmission, 123(9), 1107–1118. doi:10.1007/s00702-016-1584-8. 27311838

[ref50] *Minassian, A., Geyer, M. A., Baker, D. G., Nievergelt, C. M., O'Connor, D. T., & Risbrough, V. B., & MRS Team. (2014). Heart rate variability characteristics in a large group of active-duty marines and relationship to posttraumatic stress. Psychosomatic Medicine, 76(4), 292. doi:10.1097/PSY.0000000000000056. 24804881 PMC4062545

[ref51] Minassian, A., Maihofer, A. X., Baker, D. G., Nievergelt, C. M., Geyer, M. A., & Risbrough, V. B. (2015). Association of predeployment heart rate variability with risk of postdeployment posttraumatic stress disorder in active-duty marines. JAMA Psychiatry, 72(10), 979–986. doi:10.1001/jamapsychiatry.2015.0922. 26353072

[ref52] *Mitani, S., Fujita, M., Sakamoto, S., & Shirakawa, T. (2006). Effect of autogenic training on cardiac autonomic nervous activity in high-risk fire service workers for posttraumatic stress disorder. Journal of Psychosomatic Research, 60(5), 439–444. doi:10.1016/j.jpsychores.2005.09.005. 16650583

[ref53] Moher, D., Shamseer, L., Clarke, M., Ghersi, D., Liberati, A., Petticrew, M., … Stewart, L. A. (2015). Preferred reporting items for systematic review and meta-analysis protocols (PRISMA-P) 2015 statement. Systematic Reviews, 4(1), 1. doi:10.1186/2046-4053-4-1. 25554246 PMC4320440

[ref54] *Moon, E., Lee, S. H., Kim, D. H., & Hwang, B. (2013). Comparative study of heart rate variability in patients with schizophrenia, bipolar disorder, post-traumatic stress disorder, or major depressive disorder. Clinical Psychopharmacology and Neuroscience, 11(3), 137. doi:10.9758/cpn.2013.11.3.137. 24465250 PMC3897762

[ref55] Murison, R. (2016). The neurobiology of stress In M. Al'Absi & M.A. Flaten (Eds.), Neuroscience of pain, stress, and emotion. (pp. 29–49). London: Academic Press 10.1016/C2013-0-16065-5.

[ref56] Nagpal, M., Gleichauf, K., & Ginsberg, J. (2013). Meta-analysis of heart rate variability as a psychophysiological indicator of posttraumatic stress disorder. Journal of Trauma & Treatment, 3(2167–1222), 1000182. doi:10.4172/2167-1222.1000182.

[ref57] *Norte, C. E., Souza, G. G. L., Vilete, L., Marques-Portella, C., Coutinho, E. S. F., Figueira, I., & Volchan, E. (2013). They know their trauma by heart: An assessment of psychophysiological failure to recover in PTSD. Journal of Affective Disorders, 150(1), 136–141. doi:10.1016/j.jad.2012.11.039. 23273551

[ref58] Orr, S. P., & Roth, W. T. (2000). Psychophysiological assessment: Clinical applications for PTSD. Journal of Affective Disorders, 61(3), 225–240. doi:10.1016/S0165-0327(00)00340-2. 11163424

[ref59] *Park, J. E., Lee, J. Y., Kang, S. H., Choi, J. H., Kim, T. Y., So, H. S., & Yoon, I. Y. (2017). Heart rate variability of chronic posttraumatic stress disorder in the Korean veterans. Psychiatry Research, 255, 72–77. doi:10.1016/j.psychres.2017.05.011. 28528244

[ref60] Pichon, A., & Chapelot, D. (2010). Homeostatic role of the parasympathetic nervous system in human behavior. Hauppauge: Nova Science Publisher Inc.

[ref61] Pole, N. (2007). The psychophysiology of posttraumatic stress disorder: A meta-analysis. Psychological Bulletin, 133(5), 725. doi:10.1037/0033-2909.133.5.725. 17723027

[ref62] Ralevski, E., Petrakis, I., & Altemus, M. (2018). Heart rate variability in alcohol use: A review. Pharmacology Biochemistry and Behavior, 176, 83–92. doi:10.1016/j.pbb.2018.12.003. 30529588

[ref63] R Core Team (2018). R: A language and environment for statistical computing. R Foundation for Statistical Computing, Vienna, Austria Retrieved from https://www.R-project.org/.

[ref64] Rombold-Bruehl, F., Otte, C., Renneberg, B., Schmied, A., Zimmermann-Viehoff, F., Wingenfeld, K., & Roepke, S. (2019). Lower heart rate variability at baseline is associated with more consecutive intrusive memories in an experimental distressing film paradigm. The World Journal of Biological Psychiatry, 20(8), 662–667. doi:10.1080/15622975.2017.1372628. 29022753

[ref65] Sahar, T., Shalev, A. Y., & Porges, S. W. (2001). Vagal modulation of responses to mental challenge in posttraumatic stress disorder. Biological Psychiatry, 49(7), 637–643. doi:10.111/psyp.12027. 11297721

[ref66] Schwarzer, G. (2007). meta: An R package for meta-analysis. R News, 7(3), 40–45.

[ref67] Schwerdtfeger, A. R., Schwarz, G., Pfurtscheller, K., Thayer, J. F., Jarczok, M. N., & Pfurtscheller, G. (2020). Heart Rate Variability (HRV): From brain death to resonance breathing at 6 breaths/minute. Clinical Neurophysiology, 131 (3), 676–693. doi:10.1016/j.clinph.2019.11.03. 31978852

[ref68] Seligowski, A. V., Lebois, L. A., Hill, S. B., Kahhale, I., Wolff, J. D., Jovanovic, T., … Ressler, K. J. (2019). Autonomic responses to fear conditioning among women with PTSD and dissociation. Depression and Anxiety, 36(7), 625–634. doi:10.1002/da.22903. 31012207 PMC6602841

[ref69] Shaffer, F., & Ginsberg, J. P. (2017). An overview of heart rate variability metrics and norms. Frontiers in Public Health, 5, 258. doi:10.3389/fpubh.2017.00258. 29034226 PMC5624990

[ref70] Shaffer, F., McCraty, R., & Zerr, C. L. (2014). A healthy heart is not a metronome: An integrative review of the heart's anatomy and heart rate variability. Frontiers in Psychology, 5, 1040. doi:10.3389/fpsyg.2014.01040. 25324790 PMC4179748

[ref71] *Shah, A. J., Lampert, R., Goldberg, J., Veledar, E., Bremner, J. D., & Vaccarino, V. (2013). Posttraumatic stress disorder and impaired autonomic modulation in male twins. Biological Psychiatry, 73(11), 1103–1110. doi:10.1016/j.biopsych.2013.01.019. 23434412 PMC3648627

[ref72] *Shaikh al arab, A., Guédon-Moreau, L., Ducrocq, F., Molenda, S., Duhem, S., Salleron, J., … Vaiva, G. (2012). Temporal analysis of heart rate variability as a predictor of post traumatic stress disorder in road traffic accidents survivors. Journal of Psychiatric Research, 46(6), 790–796. doi:10.1016/j.jpsychires.2012.02.006. 22425487

[ref73] *Slewa-Younan, S., Chippendale, K., Heriseanu, A., Lujic, S., Atto, J., & Raphael, B. (2012). Measures of psychophysiological arousal among resettled traumatized Iraqi refugees seeking psychological treatment. Journal of Traumatic Stress, 25(3), 348–352. doi:10.1002/jts.21694. 22685092

[ref74] Smith, B. N., Tyzik, A. L., Neylan, T. C., & Cohen, B. E. (2015). PTSD And obesity in younger and older veterans: Results from the mind your heart study. Psychiatry Research, 229(3), 895–900. doi:10.1016/j.psychres.2015.07.044. 26210650 PMC4568132

[ref75] *Song, B. A., Yoo, S. Y., Kang, H. Y., Byeon, S. H., Shin, S. H., Hwang, E. J., & Lee, S. H. (2011). Post-traumatic stress disorder, depression, and heart-rate variability among North Korean defectors. Psychiatry Investigation, 8(4), 297. doi:10.4306/pi.2011.8.4.297. 22216038 PMC3246136

[ref76] *Spiller, T. R., Liddell, B. J., Schick, M., Morina, N., Schnyder, U., Pfaltz, M., … Nickerson, A. (2019). Emotional reactivity, emotion regulation capacity, and posttraumatic stress disorder in traumatized refugees: An experimental investigation. Journal of Traumatic Stress, 32(1), 32–41. doi:10.1002/jts.22371. 30729584

[ref77] *Tan, G., Dao, T. K., Farmer, L., Sutherland, R. J., & Gevirtz, R. (2011). Heart rate variability (HRV) and posttraumatic stress disorder (PTSD): A pilot study. Applied Psychophysiology and Biofeedback, 36(1), 27–35. doi:10.1007/s10484-010-9141. 20680439

[ref78] *Tan, G., Fink, B., Dao, T. K., Hebert, R., Farmer, L. S., Sanders, A., … Gevirtz, R. (2009). Associations among pain, PTSD, mTBI, and heart rate variability in veterans of Operation Enduring and Iraqi Freedom: A pilot study. Pain Medicine, 10(7), 1237–1245. doi:10.1111/j.1526-4637.2009.00712.x. 19818034

[ref79] Thayer, J. F., & Lane, R. D. (2007). The role of vagal function in the risk for cardiovascular disease and mortality. Biological Psychology, 74(2), 224–242. doi:10.1016/j.biopsycho.2005.11.013. 17182165

[ref80] *Thome, J., Densmore, M., Frewen, P. A., McKinnon, M. C., Théberge, J., Nicholson, A. A., … Lanius, R. A. (2017). Desynchronization of autonomic response and central autonomic network connectivity in posttraumatic stress disorder. Human Brain Mapping, 38(1), 27–40. doi:10.1002/hbm.23340. 27647521 PMC6866719

[ref81] Tolin, D.F., & Foa, E.B. (2006). Sex differences in trauma and posttraumatic stress disorder: A quantitative review of 25 years of research. Psychological Bulletin, 132(6),959–992. doi:10.1037/1942-9681.S.1.37. 17073529

[ref82] Triggiani, A. I., Valenzano, A., Ciliberti, M. A. P., Moscatelli, F., Villani, S., Monda, M., … Cibelli, G. (2017). Heart rate variability is reduced in underweight and overweight healthy adult women. Clinical Physiology and Functional Imaging, 37(2), 162–167. doi:10.1111/cpf.12281. 26211739

[ref83] *Tucker, P., Pfefferbaum, B., Jeon-Slaughter, H., Khan, Q., & Garton, T. (2012). Emotional stress and heart rate variability measures associated with cardiovascular risk in relocated Katrina survivors. Psychosomatic Medicine, 74(2), 160–168. doi:10.1097/PSY.0b013e318240a801. 22286851

[ref84] Umetani, K., Singer, D.H., McCraty, R., & Atkionson, M. (1998). Twenty-four hour time domain heart rate variability and heart rate: Relations to age and gender over nine decades. Journal of the American College of Cardiology, 31(3), 593–601. 9502641 10.1016/s0735-1097(97)00554-8

[ref85] van Aert, R. C. M., & van Assen, M. A. L. M. (2018). Correcting for publication bias in a meta-analysis with the *p*-uniform* method. Manuscript submitted for publication Retrieved from: https://osfio/preprints/bitss/zqjr92018. 10.3758/s13423-025-02812-4 PMC12948804 41760983

[ref86] Viechtbauer, W. (2010). Conducting meta-analyses in R with the metafor package. Journal of Statistical Software, 36(3), 1–48. Retrieved from https://limo.libis.be/primo-explore/fulldisplay?docid=LIRIAS1059637&context=L&vid=Lirias&search_scope=Lirias&tab=default_tab&lang=en_US&fromSitemap=1.

[ref87] Voss, A., Schulz, S., Schroeder, R., Baumert, M., & Caminal, P. (2008). Methods derived from nonlinear dynamics for analysing heart rate variability. Philosophical Transactions of the Royal Society A: Mathematical, Physical and Engineering Sciences, 367(1887), 277–296. doi:10.1098/rsta.2008.0232. 18977726

[ref88] *Wahbeh, H., & Oken, B. S. (2013a). A pilot study of clinical measures to assess mind-body intervention effects for those with and without PTSD. Alternative & Integrative Medicine, 2, 116. doi:10.4172/2327-5162.1000116. 24949490 PMC4060973

[ref89] *Wahbeh, H., & Oken, B. S. (2013b). Peak high-frequency HRV and peak alpha frequency higher in PTSD. Applied Psychophysiology and Biofeedback, 38(1), 57–69. doi:10.1007/s10484-012-9208-z. 23178990 PMC3578126

[ref90] Weathers, F. W., Keane, T. M., & Davidson, J. R. (2001). Clinician-administered PTSD scale: A review of the first ten years of research. Depression and Anxiety, 13(3), 132–156. 11387733 10.1002/da.1029

[ref91] Wickham, H., Francois, R., Henry, L., & Müller, K. (2015). dplyr: A grammar of data manipulation. R package version 0.4, 3.

[ref92] *Woodward, S. H., Arsenault, N. J., Voelker, K., Nguyen, T., Lynch, J., Skultety, K., … Sheikh, J. I. (2009). Autonomic activation during sleep in posttraumatic stress disorder and panic: A mattress actigraphic study. Biological Psychiatry, 66(1), 41–46. doi:10.1016/j.biopsych.2009.01.005. 19232575 PMC2734329

[ref93] *Woodward, S. H., Kaloupek, D. G., Schaer, M., Martinez, C., & Eliez, S. (2008). Right anterior cingulate cortical volume covaries with respiratory sinus arrhythmia magnitude in combat veterans. Journal of Rehabilitation Research & Development, 45(3), 451–463. 10.1682/JRRD.2007.06.0082. 18629753

[ref94] Yehuda, R., Southwick, S., Giller, E., Ma, X., & Mason, J. (1992) Urinary catecholamine excretion and severity of PTSD symptoms in Vietnam combat veterans. Journal of Nervous & Mental Disease. 180, 321–325. 1583475 10.1097/00005053-199205000-00006

