# Supplementary material for: Autonomic dysfunction in posttraumatic stress disorder indexed by heart rate variability: a meta-analysis
Source: Psychol Med. 2020 Aug 28;50(12):1937–48. doi: 10.1017/S003329172000207X (PMC7525781; doi:10.1017/S003329172000207X)
Supplement: Supplementary file 1 [file S003329172000207Xsup001.zip › S003329172000207Xsup009.docx]

**Supplemental table.** Characteristics of studies included in the meta-analyses

| First Author | PY | Sample  total | Sample PTSD | Type of participants | Trauma type | DSM  measure | PTSD  Measure | Categorial vs.  Correlational | HRV indices |
| --- | --- | --- | --- | --- | --- | --- | --- | --- | --- |
| Agorastos | 2013 | 15 | 7 | Veterans | Interpersonal | IV | CI | Categorial | RMSSD, SDNN, LF/HF |
| Bertram | 2014 | 46 | 22 | Veterans | Mixed | IV | CI | Categorial | HF |
| Blechert | 2007 | 55 | 23 | Others | Mixed | IV | CI | Categorial | HF |
| Brady | 2015 | 114 | n.a. | Veterans | Interpersonal | IV | CI | Correlational | SDNN, HF, LF |
| Chang | 2013 | 224 | 32 | Others | Mixed | n.a. | SR | Categorial | LF, HF, LF/HF |
| Chang | 2016 | 23 | n.a. | Others | Interpersonal | n.r. | n.r. | Correlational | RMSSD, SDNN |
| Clausen | 2016 | 24 | n.a. | Veterans | Interpersonal | IV | CI | Correlational | LF/HF |
| Cohen | 2000 | 39 | 14 | Other | Mixed | III | CI | Categorial | HF, LF |
| Dennis | 2014 | 227 | 107 | Mixed | Mixed | n.r. | CI | Categorial | SDNN, LF, HF |
| Dennis | 2016 | 219 | 99 | Mixed | Mixed | n.r. | CI | Categorial | HF |
| DePierro | 2015 | 65 | n.a. | Others | n.r. | n.a. | SR | Correlational | RMSSD, SDNN, LF, HF |
| Hausschildt | 2011 | 52 | 26 | Others | Interpersonal | IV | CI | Categorial | RMSDD, HF, LF |
| Jovanovic | 2009 | 78 | 45 | Veterans | Interpersonal | n.r. | CI | Categorial | HF |
| Kamkwalala | 2012 | 141 | 47 | Others | Mixed | IV | SR | Categorial | HF |
| Keary | 2009 | 40 | 20 | Others | Mixed | IV | CI | Categorial | HF, LF |
| Kobayashi | 2014 | 37 | 20 | Others | Mixed | IV | CI | Categorial | LF/HF |
| Lakusic | 2007 | 68 | 34 | Veterans | Interpersonal | n.r. | n.r. | Categorial | RMSSD, SDNN, HF, LF, LF/HF |
| Lee | 2012 | 125 | 37 | Veterans | Interpersonal | n.a. | SR | Categorial | RMSSD, SDNN |
| Lee | 2018 | 102 | n.a. | Others | Non-Interpersonal | 5 | SR | Correlational | HF, LF/HF |
| Liu | 2019 | 152 | 78 | n.r. | n.r. | n.r. | n.r. | Categorial | RMSSD, SDNN |
| Liverant | 2016 | 79 | n.a. | n.r. | n.r. | n.r. | CI | Categorial | HF |
| Mellman | 2004 | 19 | 9 | n.r. | n.r. | IV | CI | Categorial | LF/HF |
| Meyer | 2016 | 41 | 18 | n.r. | Mixed | IV | CI | Categorial | RMSSD, SDNN, HF, LF, LF/HF |
| Minassian | 2014 | 2235 | 120 | Others | Interpersonal | IV | CI | Categorial | RMSSD, SDNN, HF, LF, LF/HF |
| Mitani | 2006 | 22 | 10 | Others | n.r. | n.a. | SR | Categorial | LF/HF |
| Moon | 2013 | 61 | 34 | n.r. | Mixed | IV | CI | Categorial | RMSSD, SDNN, HF, LF, LF/HF |
| Norte | 2013 | 35 | 19 | n.r. | Interpersonal | IV | CI | Categorial | RMSSD |
| Park | 2017 | 141 | 68 | Mixed | Mixed | 5 | SR | Categorial | RMSSD, SDNN, HF, LF |
| Shah | 2013 | 416 | 31 | Veterans | Interpersonal | IV | CI | Categorial | LF, HF |
| Shaikh | 2012 | 21 | 11 | Others | Non-Interpersonal | IV | CI | Categorial | RMSSD, SDNN |
| Slewa-Younan | 2012 | 35 | 12 | Others | n.r. | n.r. | CI | Categorial | HF, LF, LF/HF |
| Song | 2011 | 24 | 14 | Others | n.r. | n.r. | SR | Categorial | RMSSD, SDNN, HF, LF, LF/HF |
| Spiller | 2019 | 81 | 23 | Others | Interpersonal | IV | CI | Categorial | RMSSD |
| Tan | 2009 | 28 | 16 | Veterans | Interpersonal | IV | CI | Categorial | SDNN |
| Tan | 2011 | 30 | 20 | Veterans | Interpersonal | IV | CI | Categorial | SDNN |
| Thome | 2016 | 98 | 57 | n.r. | n.r. | IV | SR | Categorial | RMSSD, HF, LF |
| Tucker | 2012 | 45 | 13 | Others | Non-Interpersonal | IV | CI | Categorial | HF, LF, LF/HF |
| Van Male | 2000 | 30 | n.a. | n.r. | n.r. | n.r. | CI | Categorial | HF |
| Wahbeh | 2013 | 30 | 15 | Veterans | Interpersonal | IV | CI | Categorial | HF |
| Wahbeh | 2013 | 81 | 52 | Veterans | Interpersonal | IV | CI | Categorial | HF, LF, LF/HF |
| Wisco | 2015 | 10 | n.a. | n.r. | n.r. | n.r. | CI | Categorial | HF |
| Woodward | 2009 | 35 | 22 | Others | Mixed | IV | CI | Categorial | HF |
| Woodward | 2008 | 77 | 40 | Veterans | Interpersonal | IV | CI | Categorial | HF |

Note. PY = Publication year, CI = Clinical Interview, SR = Self-report, n.r. = not reported, n.a. = not applicable
