# Supplementary figures and images for: Autonomic dysfunction in posttraumatic stress disorder indexed by heart rate variability: a meta-analysis
Source: Psychol Med. 2020 Aug 28;50(12):1937–48. doi: 10.1017/S003329172000207X (PMC7525781; doi:10.1017/S003329172000207X)

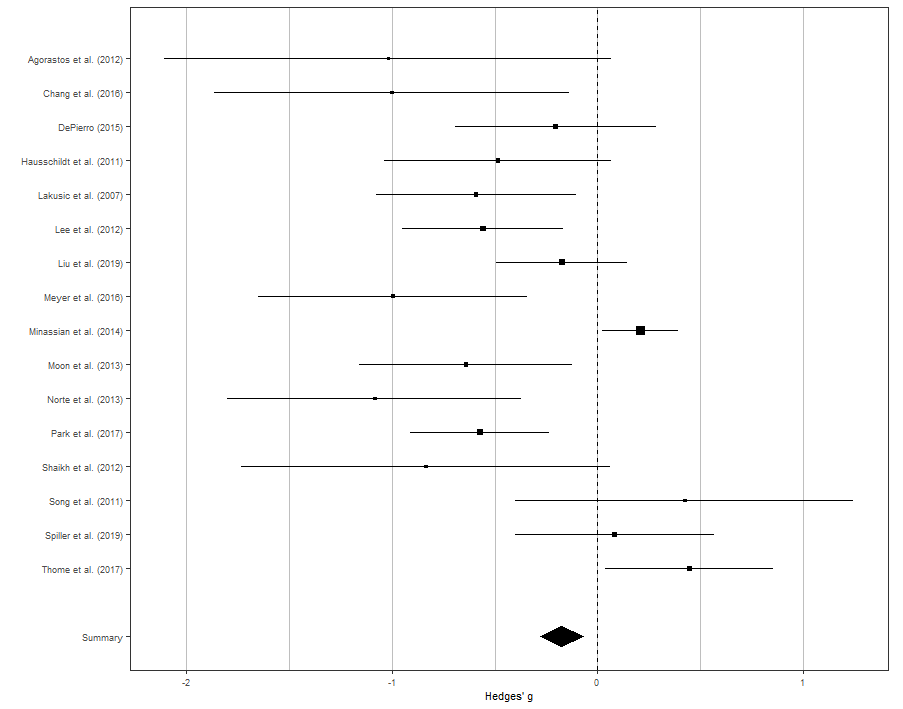

Supplement: Supplementary file 1 [file S003329172000207Xsup001.zip › S003329172000207Xsup001.tiff]

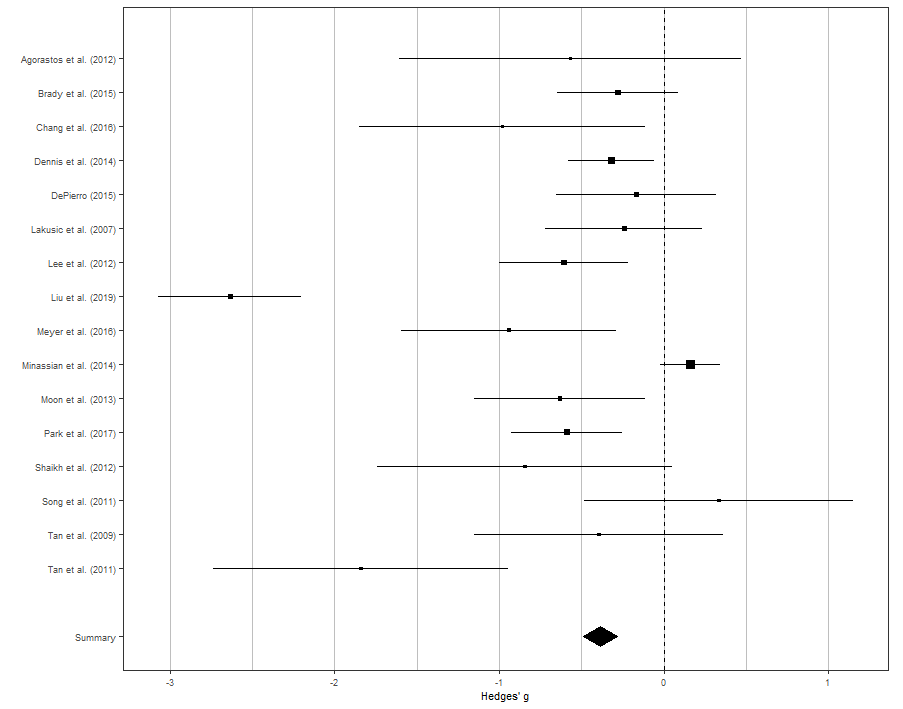

Supplement: Supplementary file 1 [file S003329172000207Xsup001.zip › S003329172000207Xsup002.tiff]

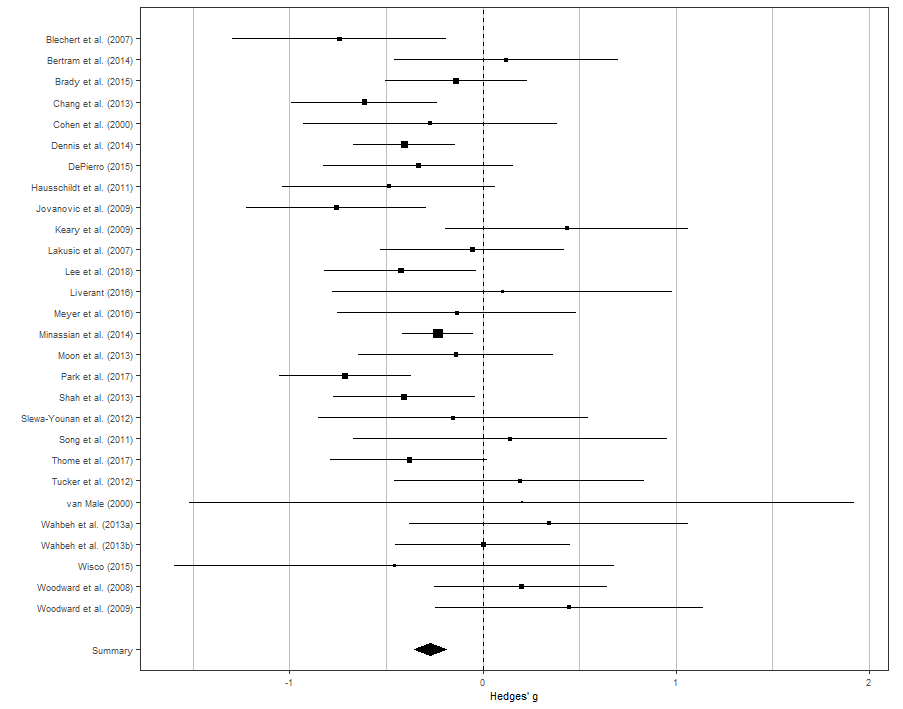

Supplement: Supplementary file 1 [file S003329172000207Xsup001.zip › S003329172000207Xsup003.tiff]

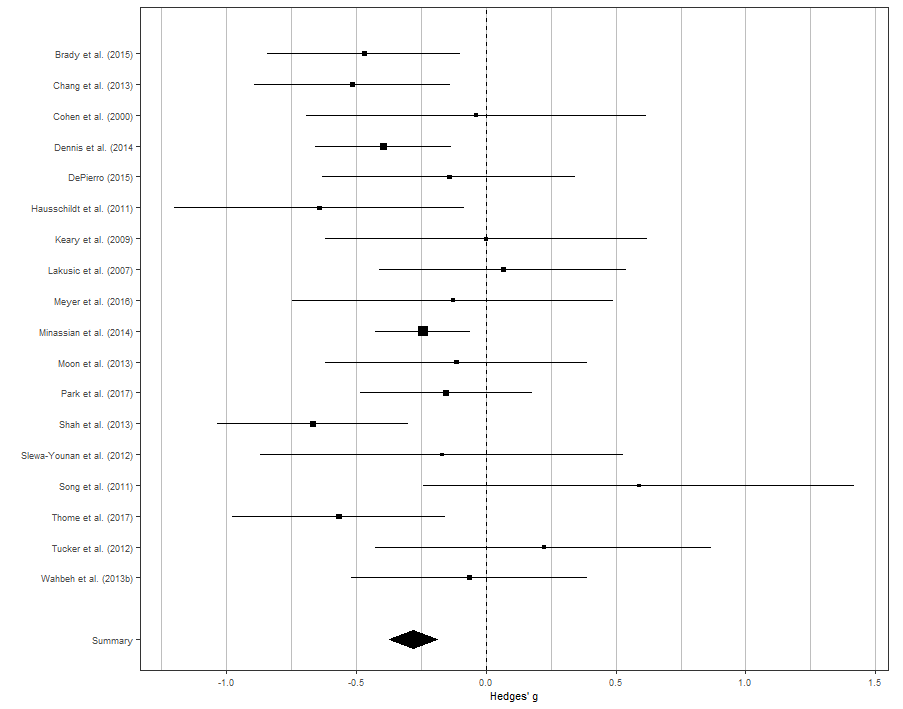

Supplement: Supplementary file 1 [file S003329172000207Xsup001.zip › S003329172000207Xsup004.tiff]

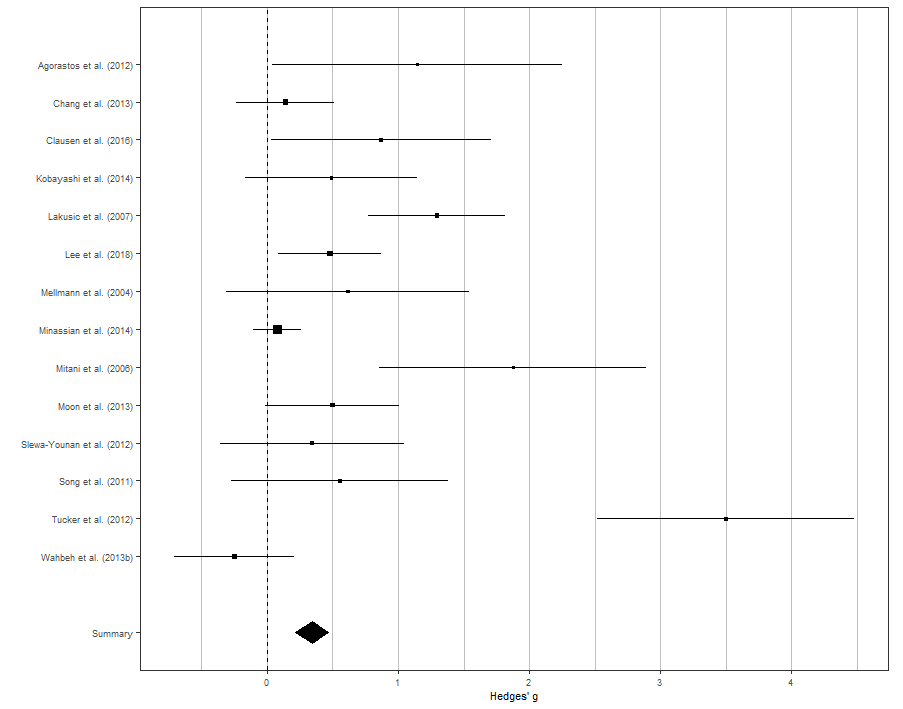

Supplement: Supplementary file 1 [file S003329172000207Xsup001.zip › S003329172000207Xsup005.tiff]

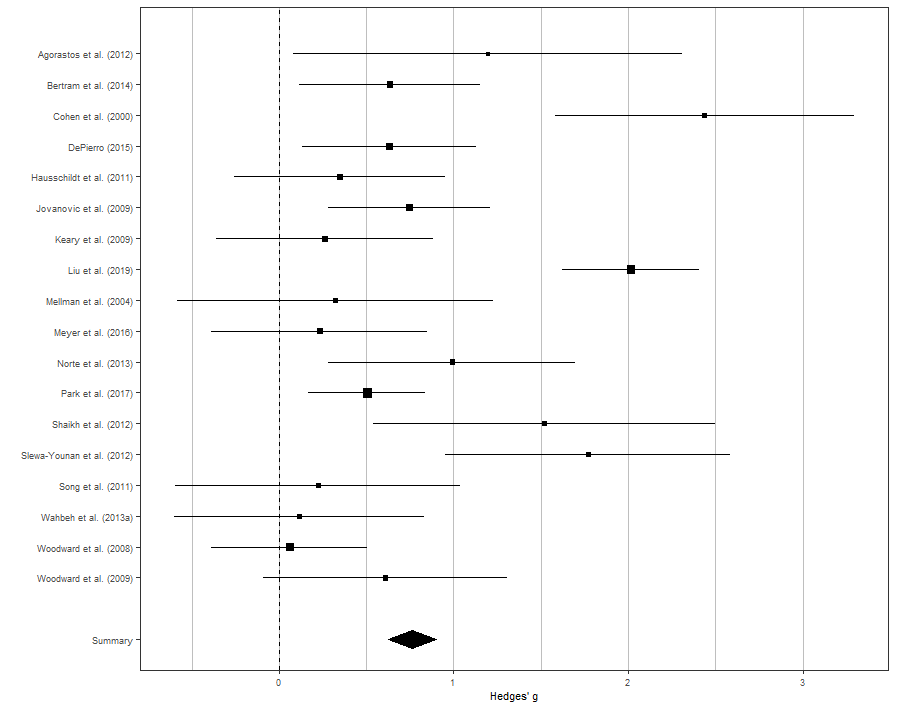

Supplement: Supplementary file 1 [file S003329172000207Xsup001.zip › S003329172000207Xsup006.tiff]

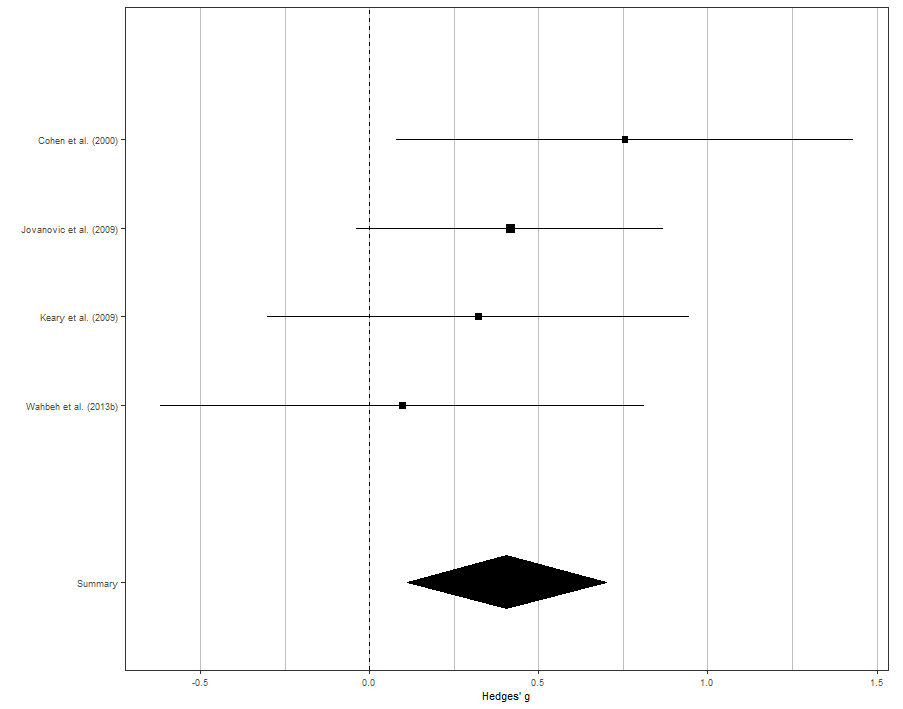

Supplement: Supplementary file 1 [file S003329172000207Xsup001.zip › S003329172000207Xsup007.tiff]

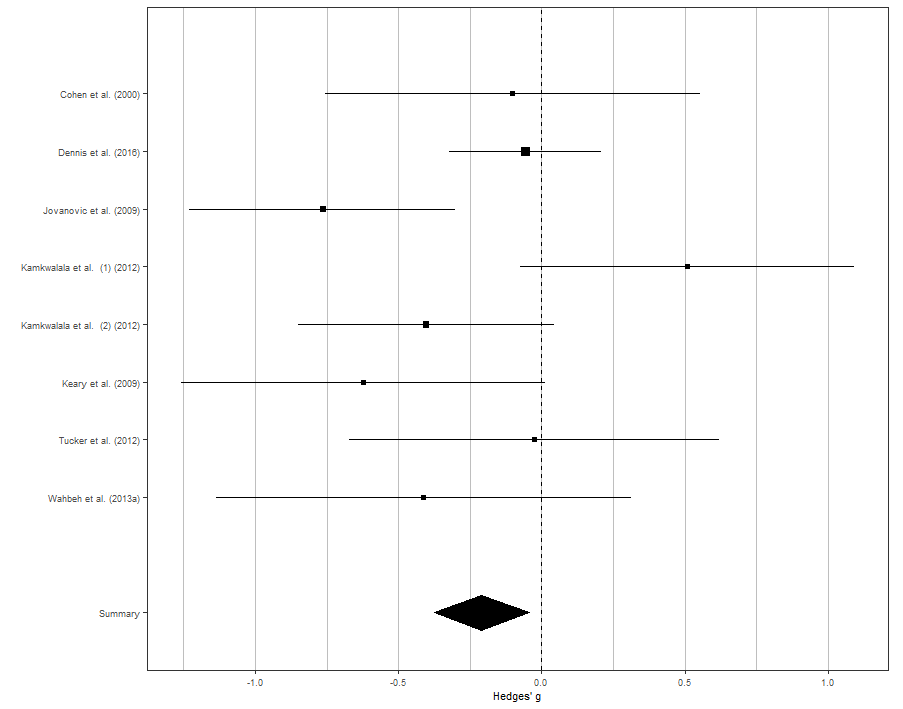

Supplement: Supplementary file 1 [file S003329172000207Xsup001.zip › S003329172000207Xsup008.tiff]
